# Current Hospital Topics

**Published:** 1906-06-16

**Authors:** 


### HOSPITAL ADMINISTRATION.

### CONSTRUCTION AND ECONOMICS.

### CURRENT HOSPITAL TOPICS.

**Hospital Sunday.**

Sunday the 17th instant being Hospital Sunday in London, we have issued this year a Special Hospital Sunday Supplement bearing that date, which will be distributed in the places of worship throughout London on behalf of the hospitals. The Ven. Archdeacon Sinclair, in sending his thanks for his copy, describes it as "the most interesting, valuable, and effective number." Any of our readers may obtain a copy on application to the publisher.

**Ambulances and Hospitals in London.**

At the present time there is an undoubted need for some system which will make it possible for an ambulance to be at once obtained to convey accident cases to the hospitals. Our attention has been called to the case of a page boy who fell through a skylight to the basement and injured himself severely. When the services of a private practitioner were obtained he advised the friends to telephone to the hospital for an ambulance. This the hospital was unable to supply, and no ambulance was available because the police can only supply one in those cases where accidents happen in the streets. It would thus appear that there is no ambulance provision of any kind for the conveyance of accidents which may happen in houses, to the hospitals. We have no doubt that the County Council will take steps to remedy this defect so soon as Parliament has passed the Bill now under consideration which was down for its third reading in the House of Commons this week. Meanwhile this case illustrates the urgent necessity for the action taken by the London County Council in regard to ambulance provision in the metropolis.

**Too Many Secretaries?**

It has been pointed out to us that Mr. C. J. Michod, who was referred to in this column last week, had nothing whatever to do with the Festival Dinner of Charing Cross Hospital, though he wrote to the papers as Secretary of the Charing Cross Hospital Emergency Fund. The success of that festival dinner was largely owing, as the American Ambassador pointed out, to the self-denying devotion of Mrs. Arthur Paget, whose sufferings and patience have won for her the sympathy of two continents. The organisation and general work connected with the dinner were carried through by Lord Kilmorey, Mr. Arthur Reade, the Secretary, and Mr. Wooldridge, the Assistant Secretary of Charing Cross Hospital, to all of whom much credit is due. We regret the error of attributing the credit to Mr. Michod for the success of this festival dinner, but where there are several secretaries representing one institution, and one of them writes with apparent authority to the papers about its work, it is not always easy to apportion the credit in due proportion to the work done. No doubt Lord Kilmorey, who has a very uphill task before him in connection with Charing Cross Hospital, will speedily carry through any reorganisation of the machinery which his experience may show him to be necessary.

**Hospital Authorities and Hospital Complaints.**

The Birmingham newspapers during the last few weeks have been rather full of matter bearing such titles as "Another Hospital Inquiry," and "Alleged Neglect at the General Hospital." In one case the complaint was made by a father in regard to his child whom he took to the hospital suffering from burns. In another the patient was a poor woman whom a local practitioner sent to the hospital on the ground that only an operation could save her life. The last patient was accompanied by Nurse E. Bodell, of the Salvation Army, and a lady who had interested herself in the case. After a delay tne child was admitted, but the woman after waiting two hours was sent away on the ground that there was no bed vacant. The Committee of the General Hospital hold the officials immediately concerned to be blameless, but from the public point of view repeated cases of the character named must undermine confidence, and we hope that steps may be taken to prevent any further complaints of the kind. Many people, no doubt, are inclined to be impatient when ill, but the constant handling of hundreds of out-patients, and the necessity to make haste on the part of the officers dealing with these cases, may tend to cause forgetfulness of the claim which all patients must have upon the officials of a hospital for the most considerate and kindly treatment at all times. The inhabitants of Birmingham on the whole probably give in greater numbers to the support of the hospitals than the people of any other city, and they have a right to demand from the hospital authorities that cases of complaint shall be conspicuous by their absence.

**The Need of Amalgamation.**

The King's Hospital Fund has done good service by using its influence to secure the amalgamation of certain small special hospitals so as to provide one modern hospital of adequate proportions and of the highest efficiency for the treatment of in- and out-patients. In the provinces, from time to time, the desirability of amalgamating the hospitals in certain centres of population has been made apparent, and the absence of a central fund to bring it about has often been felt. At Bournemouth at the present time there is great need for the exercise of wisdom and authority to secure the amalgamation of the Royal Victoria and the Royal Boscombe and West

Hants Hospitals. The former institution has outgrown the provision which its site allows, and in consequence, the cost of its maintenance compares unfavourably with the relatively low rates maintained at the Boscombe Hospital, which is intelligently and economically administered. The Royal Victoria Hospital has had a dispensary situated midway between that hospital and the Boscombe hospital. At the moment there is a proposal to erect a large new out-patient department in lieu of the dispensary on a new site midway between the two hospitals, and to erect an in-patients' block in connection with it. If this proposal is proceeded with, it will mean, in fact, that Bournemouth will be asked to maintain three general hospitals, a number out of all proportion to the needs and requirements of the population. We hope that the people of Bournemouth will be wise enough to bring pressure to bear upon the hospital authorities concerned, with a view to secure an amalgamation of the two existing hospitals, so that Bournemouth may have one modern up-to-date and efficient general hospital. We would suggest that the questions at issue should be referred to a committee of three representatives, one of whom should be appointed by the Royal Victoria Hospital, the Boscombe Hospital, and the Hospital Sunday and Saturday Funds respectively.

### A Municipal and Rate-supported Hospital!

One advantage to the public of having a hospital in charge of a responsible superintendent is that, as the head of the administration, if he be a man of parts he can fearlessly state in his annual report the defects of his hospital. The Cincinnati Hospital is a municipal institution; the average number of in-patients during 1905 was 513, and attached to it are a branch hospital for tuberculous cases and a branch hospital annex for infectious diseases. The superintendent reports that the former "is rapidly becoming a mere convenience for the isolation of persons in the last stage of consumption rather than a hospital for treatment and cure. The very object of opening the branch hospital is rapidly being rendered nugatory by the over-crowding of patients, as it prevents those suffering from tuberculosis from getting the benefit of the most scientific, up-to-date treatment. If people in robust health were crowded together in any building as they are at the branch hospital, it would not take the grim reaper long to depopulate such a building. Two and sometimes three people afflicted with consumption, are crowded into a space that should be occupied by not more than one person suffering with that disease. So bad is the atmosphere of the wards that it is not only highly detrimental to the afflicted, but is a menace to those who minister to them and are compelled to breathe the disease-laden atmosphere." Of the branch hospital annex he reports that the authorities "had been compelled to take patients from the county gaol and city work-house and domicile them in the same ward with decent respectable citizens; all of which has occasioned much complaint, to say nothing of the great injustice of compelling decent law-abiding citizens to commingle with criminals and some of the lowest of the low." We most strongly commend the courage of the superintendent who exposes grave abuses like those here recorded as existing in the hospital under his charge. The Cincinnati Hospital is a municipal institution, and the state of affairs here revealed is one more evidence of the risks which the British public might incur were the hospitals in this country to become municipalised. We are glad to know that the people of Cincinnati are taking active steps to secure the erection of a new up-to-date and modern hospital for the reception of the sick of all classes in their city.

